# Rapid 3D Measurement of Tire–Pavement True Contact Texture and Its Implications for Skid Resistance

**DOI:** 10.3390/ma19091856

**Published:** 2026-04-30

**Authors:** Tursun Mamat, Siyi Cheng, Li Xu, Shenqing Xiao, Chunguang He

**Affiliations:** 1 School of Transportation and Logistics Engineering, Xinjiang Agricultural University, Urumqi 830052, China; tursun@xjau.edu.cn (T.M.); 17395654919@163.com (S.C.); hechunguang@xjau.edu.cn (C.H.); 2 Intelligent Transportation Engineering Research Center, Xinjiang Agricultural University, Urumqi 830052, China; 3 Xinjiang Key Laboratory of Transportation and Logistics Engineering, Xinjiang Agricultural University, Urumqi 830052, China; 4 School of Machinery and Automation, Wuhan University of Science and Technology, Wuhan 430081, China; 19904638828@163.com

**Keywords:** asphalt pavement, tire-pavement contact, three-dimensional texture, pavement skid resistance, pavement texture

## Abstract

**Highlights:**

- A rapid 3D measurement method for tire–pavement contact texture has been developed.
- Higher loads increase the real contact area, penetration depth, and fractal dimension.
- Penetration depth strongly correlates with skid resistance, whereas fractal dimension does not.
- These findings enable precise predictions of interlocking effects under real conditions.

**Abstract:**

Accurate characterization of the true tire–pavement contact state is essential for understanding pavement friction; yet conventional texture indicators and nominal contact assumptions cannot directly represent the actual interfacial interaction between rubber and pavement. This study proposes a rapid and non-destructive method for measuring three-dimensional tire–pavement true contact texture under different loads. A materials testing system was used to apply controlled loads to a rubber pad–carbon paper–pavement assembly, and the resulting imprints were combined with three-dimensional laser profilometer data and support-curve-based slicing to determine the real contact area ratio, penetration texture depth, and self-affine fractal dimension. Tests on nine asphalt pavement samples under loads from 5 to 20 kN showed that the real contact area ratio increased with load but remained below 40% at 20 kN. The predicted contact area from the reconstructed 3D texture agreed well with the imprint-based results, with an absolute error not exceeding 2.59%. Penetration texture depth showed a stronger relationship with skid resistance than fractal dimension. The proposed method provides a practical means of capturing effective tire–pavement contact parameters and offers useful inputs for laboratory-based skid resistance evaluation and texture-informed friction modeling.

## 1. Introduction

Tire–pavement contact typically describes the interaction between tires and pavements. For a constructed pavement, its surface needs to maintain sufficient roughness throughout its long-term service life to provide friction. The pavement surface texture also affects various performance factors, including skid resistance, lateral stability, noise levels, drainage capacity, fuel consumption, and driving comfort [1]. The pavement friction is the fundamental source of vehicle braking capability. In the future, intelligent transportation vehicles must continuously sense the pavement friction levels in real-time to make accurate decisions related to braking, steering, and other driving maneuvers [2]. However, the mechanisms underlying sliding and rolling friction remain a significant scientific challenge, and a unified model has yet to be developed. Persson [3] and Luo et al. [4] developed a friction model based on the viscoelastic behavior of rubber on rough surfaces at different scales (from micro to macro), which has led to increased research interest in tire–pavement contact friction.

The Real Area of Contact (RAC) is a crucial parameter commonly used in assessing tire–pavement contact conditions. In fact, all tribological phenomena occur within the RAC. This area generally typically represents a small portion of the nominal contact area, and quantifying it can enhance our understanding of contact physics [5]. The nominal (or apparent) contact area is a readily measurable parameter. To simplify contact friction models, many pavement mechanics models typically simplify the tire–pavement contact area into regular shapes such as circles or rectangles [6,7]. However, these simplifications often do not adequately capture the complexity of real friction systems. For measurements of the RAC, imprint copying serves as an incipiently straightforward method for obtaining the tire–pavement contact imprints [8]. Some researchers have employed transparent medium methods, such as transparent rubber, glass, and larger-sized platforms [9], in conjunction with contact state images for mechanical analysis. Since the introduction of the Fuji pressure-sensitive film measurement system in the 1980s, its application has become widespread across numerous industries [10]. Zhang et al. [11] employed the pressure-sensitive technique to measure the contact area between the tire and the pavement under different pressures, achieving a measurement resolution of 0.016 mm^2^. Dubois et al. [12] attempted to utilize Tekscan pressure sensors as a more convenient testing method to measure pressure distribution on the pavement surfaces with different roughness levels. These testing techniques offer valuable strategies for assessing the tire–pavement contact states. Nonetheless, all of these testing methods have somewhat interfered with the contact state of the friction system by applying surface treatments or introducing additional materials. Kriston et al. [13] suggested a non-destructive testing method utilizing a CT scanning technique to examine the contact state between pavement, snow, and rubber materials at a microscopic scale. Processing CT images of contact areas formed by rubber compounds of varying hardness levels facilitates the visualization and accurate measurement of the RAC, thereby promoting the application of micro-CT in contact mechanics. Guo et al. [14] and Li et al. [15] developed an onboard 3D laser profilometer capable of conducting robust non-destructive testing of pavement surfaces at speeds ranging from 20 to 80 km/h. It continuously collects 3D pavement texture data with an accuracy of 0.01 mm, which helps accurately quantify processes such as tire–pavement interaction and texture wear.

Additionally, researchers have proposed various theoretical estimation methods for tire–pavement contact area. Von Meier et al. [16] developed a mathematical empirical algorithm for the envelope profile of surface texture based on the condition that the second derivative of surface texture is less than the stiffness of tire rubber. Chen et al. [17] applied the Hilbert–Huang Transform (HHT) theory to analyze the combined intrinsic modes of surface structures in mixtures and computed the envelopes of these surface structures. Yun et al. [18] utilized a plasticine indentation method to characterize the contact state between tires and triangular prism grooves during automobile travel by examining the morphology of pressed plasticine. This led to the determination of the effective envelope profile depth of tire–pavement contact. Nonetheless, the use of clay as a tire–pavement contact medium greatly compromised the real contact state. Kanafi and Tuononen [19] indicated that the roughness of the top 20% of a pavement’s surface at wavelengths less than 1 mm is highly correlated with the friction coefficient. Pinnington [20] proposed a universal algebraic model covering the entire wavelength range of tire–pavement contact. They established a theoretical numerical model for tire–pavement contact profiles by utilizing measured pavement profile data ranging from 0.1 mm to 100 m. The model employed truncated Fourier series to characterize the particle shapes and envelopes of particle peaks as Weierstrass–Mandelbrot functions.

Currently, a large amount of engineering test data has not yet demonstrated the intrinsic relationship between skid resistance performance and texture characteristics. This may be due to the fact that indicators characterizing surface texture, such as mean profile depth (MPD), mean texture depth (MTD), and other multiscale texture indicators (including amplitude, spacing, and composite parameters [21]), merely describe the texture characteristics. These indirect evaluation methods weaken the non-complete contact between the tread rubber and the pavement surface, leading to suboptimal skid resistance prediction models.

Although apparent contact geometry is often used in pavement friction analysis, the true tire–pavement contact state is governed by multiscale surface roughness, local deformation of rubber, and environmental conditions [22]. As a result, relying only on nominal contact area or conventional texture descriptors may lead to an incomplete representation of the actual interfacial interaction responsible for skid resistance [21]. This limitation becomes more critical under service conditions involving speed, water film, and complex tire-road interaction, where the discrepancy between apparent and true contact can substantially affect friction-related assessment. Therefore, a method capable of directly extracting physically meaningful contact parameters from the pavement–rubber interface is needed to improve the interpretation of skid resistance.

Therefore, the central hypothesis of this study is not merely that contact area or penetration depth increases with load, but that a rapid measurement method combining imprint extraction, digital image processing, and 3D texture reconstruction can capture true contact parameters that are more physically representative of pavement skid resistance than conventional macroscopic texture indicators alone. Given the complexity and variability of real tires and tread patterns, a natural rubber pad was used in this study as a simplified surrogate to develop and verify the measurement framework under controlled laboratory conditions. Based on 3D laser profilometer data and imprint-derived contact information, this study aims to quantify the real contact area ratio, penetration texture depth, and self-affine fractal dimension under different loads and to examine their relevance to pavement skid resistance. The findings are expected to provide methodological support for contact-based texture characterization and for future development of texture-informed skid resistance evaluation models.

## 2. Materials and Experimental Methods

### 2.1. Material Properties

#### 2.1.1. Design of Common Pavement Rutting Plates

Nine rutting plate samples were designed based on conventional pavement types. The AC series (continuous gradation asphalt concrete pavement) consists of three rutting plate samples, designated AC10, AC13, and AC16. The SMA series (Stone Mastic Asphalt pavement) includes three rutting plate samples, labeled SMA10, SMA13, and SMA16. The OGFC series (Open-Graded Friction Course asphalt pavement) contains three rutting plate samples, identified as OGFC10, OGFC13, and OGFC16.

**Figure 1:**
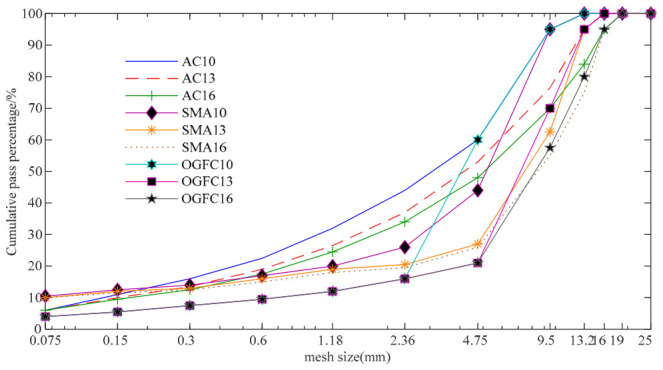
Design gradation curves of aggregates for various types of rutting plates.

The coarse and fine aggregates for asphalt mixtures were selected in accordance with the “Test Method of Aggregates for Highway Engineering” (JTG E42-2005). The main technical indicators of these aggregates are summarized in Table 1. Figure 1 presents the design gradation curves of aggregates for rutting plates, all of which fall within the upper and lower limits.

#### 2.1.2. Rubber Pad

Vehicle tires are generally designed with different materials and treads to fulfill specific driving requirements. Given the complexity of the interaction between the tire rubber and the pavement surface, this study employed commonly available rubber pads (natural rubber) to simulate their direct contact. The tire type is not the focus of this study. The performance parameters for the rubber pads are presented in Table 2.

### 2.2. Experimental Methods

#### 2.2.1. 3D Reconstruction of Pavement Surface

**Figure 2:**
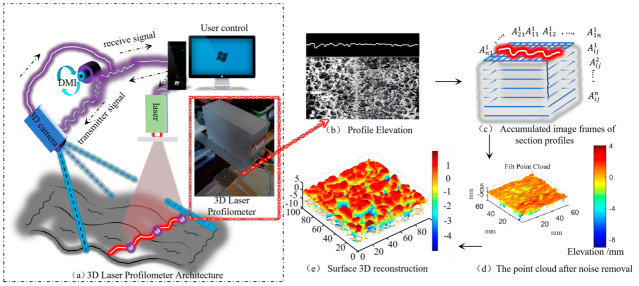
Workflow of collecting surface information of the targets using a 3D laser profilometer: (**a**) 3D laser profilometer architecture; (**b**) profile elevation; (**c**) accumulated image frames of section profiles; (**d**) point cloud after noise removal; and (**e**) surface 3D reconstruction.

This study employed a 3D laser profilometer (see Figure 2a) to obtain 3D information about the pavement surface (Shenzhen SSSZNI, Shenzhen, China). The 3D laser profilometer consists of a high-speed 3D camera, a line laser emitter, an axis encoder (Distance Measuring Instrument, DMI), a Gigabit Ethernet signal transmission line, and a control center. The adoption of a 3D laser profilometer to collect initial surface information of samples can ensure the reliability of data sources. The operational principles of this data acquisition system are detailed in the existing literature and will not be elaborated upon [15]. The rutting plate serves as the subject of this study. The 3D reconstruction primarily involves four steps: (1) obtaining the elevation data of the target profile; (2) acquiring accumulated image frames of the target profile; (3) gathering the target point cloud and applying filtering to reduce noise; (4) performing 3D surface reconstruction based on the point cloud data.

**Figure 3:**
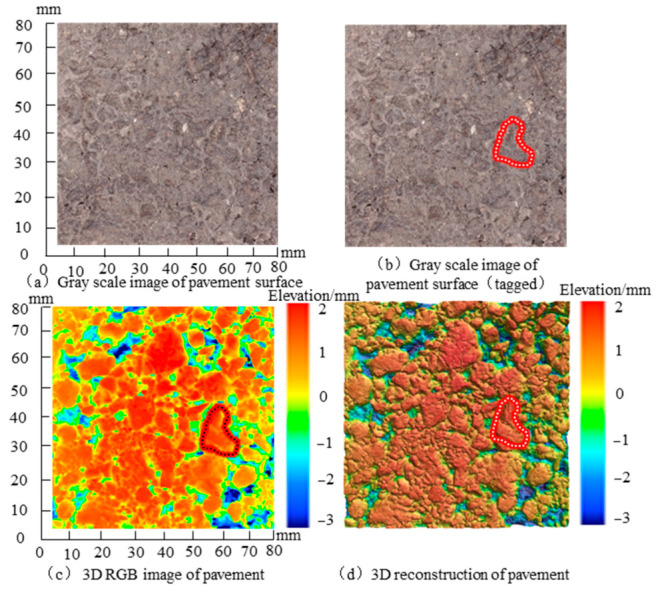
3D texture reconstruction for the asphalt pavement: (**a**) gray-scale image of the pavement surface; (**b**) gray-scale image with the selected local texture region marked by a red dashed contour; (**c**) 3D RGB image of the pavement surface; and (**d**) 3D reconstruction of the pavement surface. The red dashed contour indicates the same local texture region used for visual comparison among the different image representations.

The acquired point cloud data contains relative elevation information (x, y, and z) with an accuracy of up to 10 μm. It should be noted that the grayscale images of the pavement surface captured by the 3D camera were derived from elevation data, which prevents image distortion caused by lighting effects when using traditional cameras for direct photography. This approach facilitated data collection from a central area measuring 80 × 80 mm of the AC13 sample, which allowed for the acquisition of multiple pavement surface parameters, including elevation grayscale (0–255) information, 3D RGB data, and 3D reconstruction information, as illustrated in Figure 3.

#### 2.2.2. Tire–Pavement Contact Imprint Extraction Test

**Figure 4:**
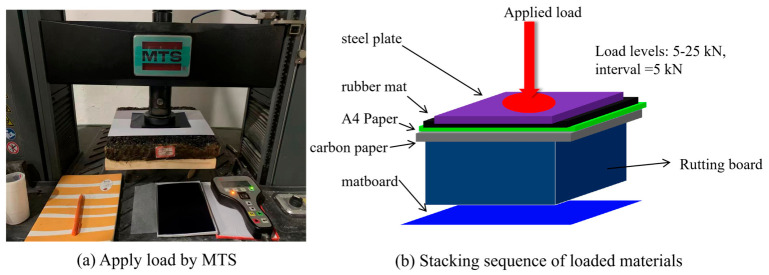
Testing of contact between rubber pad and asphalt surface under different loads: (**a**) application of load by MTS; (**b**) stacking sequence of loaded materials.

The testing equipment used is the MTS E45.105 material testing machine, referred to as MTS hereafter, as depicted in Figure 4a. The technical requirements of the measurement system comply with the standards GB/T16825.1 and ASTM E4. The strain measurement system adheres to GB/T 12160 and ASTM E83. The main operational steps for extracting contact imprints are illustrated in Figure 4. (1) Position a custom flat wooden board (300 mm × 300 mm × 25 mm) on the base of the testing machine’s “lower space,” which is automatically leveled to prevent eccentric pressure. Sequentially stack the rutting plate, A4 paper, carbon paper, plain paper, and rubber pad on the board, as illustrated in Figure 4b. The parameters of equipment and materials are provided in Table 3.

The selected loading levels were intended to cover a controlled contact-pressure range relevant to laboratory simulation of tire–pavement interaction. Based on the circular loading area with a diameter of 10 cm, the applied loads of 5, 10, 15, 20, and 25 kN correspond to nominal pressures of 0.64, 1.27, 1.91, 2.55, and 3.18 MPa, respectively. In particular, the 5 kN condition is close to the commonly referenced wheel pressure level of approximately 0.7 MPa under the standard axle load, whereas the higher loads were used to investigate the evolution of contact behavior under increased local compression.

Carbon paper was adopted as the imprinting medium because it is thin, inexpensive, and easy to handle, thereby reducing disturbance to the contact interface compared with thicker pressure-sensitive interlayers. A4 paper was used as a flat carrier for imprint transfer and scanning. Natural rubber was employed as a simplified surrogate material to represent the deformable nature of tire tread in a controlled laboratory framework; however, it does not reproduce the full composition, geometry, inflation, or dynamic behavior of an actual tire. The 1 min holding time was selected to ensure stable imprint formation and to reduce the influence of transient loading fluctuations before unloading.

The imprinting procedure was performed in four steps: (1) Place the natural rubber specimen, carbon paper, and A4 carrier paper beneath the circular loading plate, and ensure that the loading area is properly aligned. (2) Apply forces ranging from 5 to 25 kN to a circular loading area with a diameter of 10 cm, and hold the specified maximum load for 1 min before unloading. The applied loads are detailed in Table 4. (3) Record the beam displacement and load application status. (4) Extract the carbon paper and insert it into the printer to capture the imprint.

Although a 25 kN loading condition was attempted in the test program, the paper interlayer used for imprint extraction ruptured under this condition, which prevented reliable acquisition of the contact imprint. Therefore, only the results from 5 to 20 kN were included in the subsequent quantitative analysis.

### 2.3. Calculation of Texture Characterization Parameters

#### 2.3.1. Calculation of Real Contact Ratio

**Figure 5:**
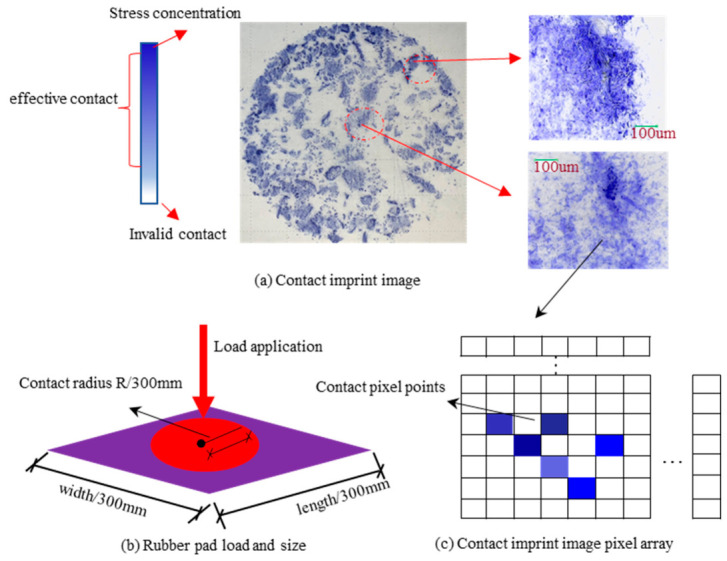
Contact imprint images.

As illustrated in Figure 5a, the contact imprint image obtained from the printer is a color image that includes brightness and color information. Each pixel in an RGB (Red/Green/Blue) image contains data for the three colors: red, green, and blue, as depicted in Figure 5c. Each color channel is one byte in size, resulting in a total of three bytes per pixel (24-bit binary). Grayscale images convey brightness information, wherein each pixel has a single grayscale level (ranging from 0 to 255) and a size of one byte (8-bit binary).

Each imprint image comprises two components: the contact area and the background. As observed from the direct scan of the imprint in Figure 5a, the background is grayish, and the pixel grayscale levels are not zero, which complicates the differentiation between the two. A straightforward method to address this issue is to segment the background from the contact area using a thresholding method. This study first performed statistical analysis of the grayscale histogram for color images to determine the optimal segmentation threshold, t. The grayscale image is converted into a binary image using the threshold t, and the corresponding transformation function is expressed as:

(1) $$g(x,y)=\left\{\begin{array}{c} 0\\ 255 \end{array}\right.\begin{array}{c} ,f(x,y)\leq t\\ ,f(x,y)>t \end{array}$$

where g(x,y) denotes the grayscale value after binarization of the pixel, f(x,y) represents the grayscale value before binarization of the pixel, and t denotes the grayscale threshold (threshold value).

Theoretically, the target in the image is distributed in the lower grayscale levels, while the background is predominantly lighter and falls into the higher grayscale levels. When segmenting the image, a threshold that is too high may capture unnecessary parts, while a threshold that is too low could result in the loss of areas of interest. Effective and commonly used methods for determining the threshold include the minimum threshold method, maximum error threshold method, and optimal threshold method. This study employed the maximum variance thresholding method for background and target segmentation.

##### Theory of Maximum Variance Thresholding Method

This method assumes that a specific threshold in the grayscale histogram divides the data into two groups, and the threshold is determined when the variances of the two groups are maximized. It is assumed that an arbitrary image has grayscale levels ranging from 1 to m, where the number of pixels with a grayscale value of i is $n_{i}$. The total number of pixels can be expressed as:

(2) $$N=\sum_{i=1}^{m}n_{i}$$

The probability for each grayscale value is:

(3) $$P_{i}=\frac{n_{i}}{N}$$

To determine the threshold T, the image grayscale values are divided into two groups, $C_{0}=\left\{1\sim T\right\}$ and $C_{1}=\left\{1+T\sim m\right\}$, the probabilities for each group are:

The occurrence probability of $C_{0}$ is:

(4) $$w_{0}=\sum_{i=1}^{T}P_{i}=w(T)$$

The occurrence probability of $C_{1}$ is:

(5) $$w_{1}=\sum_{i=T+1}^{m}P_{i}=1-w_{0}$$

The mean of $C_{0}$ is:

(6) $$\mu_{0}=\sum_{i=1}^{T}\frac{iP_{i}}{w_{0}}=\frac{\mu (T)}{w(T)}$$

The mean of $C_{1}$ is:

(7) $$\mu_{1}=\sum_{i=T+1}^{m}\frac{iP_{i}}{w_{1}}=\frac{\mu -\mu (T)}{1-w(T)}$$

where $\mu =\sum_{i=1}^{m}iP_{i}$. represents the overall mean grayscale value of the image, while $\mu (T)=\sum_{i=1}^{T}iP_{i}$ indicates the mean grayscale value at threshold T. Thus, the mean of the sampled grayscale is:

(8) $$\mu =w_{0}\mu_{0}+w_{1}\mu_{1}$$

The grayscale variance between the two groups can be expressed as:

(9) $$\delta^{2}(T)=w_{0}{\left(\mu_{0}-\mu \right)}^{2}+w_{1}{\left(\mu_{1}-\mu \right)}^{2}=w_{0}w_{1}(\mu_{1}-\mu_{0})=\frac{{\left[\mu w(T)-\mu (T)\right]}^{2}}{w(T)[1-w(T)]}$$

The $T^{*}$ that satisfies $\delta^{2}(T)$ = $\max\delta^{2}(T)$ is the threshold of maximum variance.

Substituting Equation (9) into Equation (1) yields the real contact ratio λ:

(10) $$\lambda =\sum_{i=1}^{m}\sum_{j=1}^{n}P_{ij}/m\times n$$

where $P_{ij}$ represents the points with a pixel value of 255 in the two-dimensional pixel matrix.

#### 2.3.2. Calculation Methods for Pavement Surface Texture Parameters

**Figure 6:**
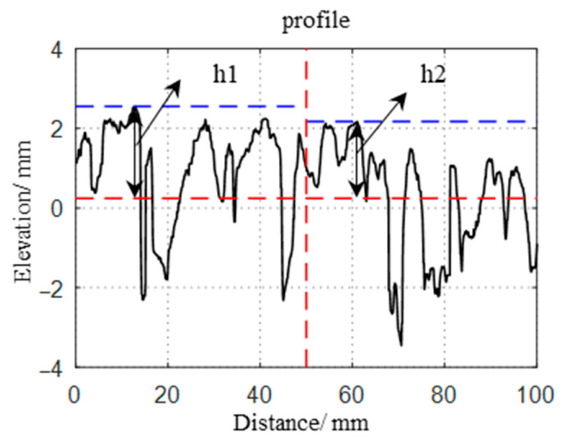
Calculation diagram for the MPD. The vertical red dashed line divides the evaluation length into two equal segments; the horizontal blue dashed lines indicate the maximum peak levels (h1 and h2) of each segment; and the horizontal red dashed line represents the mean profile level.

**Mean profile depth (MPD)**. The calculation diagram for the MPD (in mm) is illustrated in Figure 6.

**Mean texture depth (MTD)**. The calculation for the mean texture depth (in mm) is as follows:

(11) $$MTD=\frac{1}{M\times N}\sum_{i=1}^{M}\sum_{j=1}^{N}\left[\max\left\{Z\left(x_{i},y_{j}\right)\right\}-Z\left(x_{i},y_{j}\right)\right]$$

where *M* and *N* represent the total number of data points along the *X* and *Y* axes, respectively; $Z\left(x_{i},y_{j}\right)$ denotes the elevation value of the pavement surface at the coordinate $\left(x_{i},y_{j}\right)$; and $\max\left\{Z\left(x_{i},y_{j}\right)\right\}$ is the maximum elevation value within the selected evaluation area.

**Figure 7:**
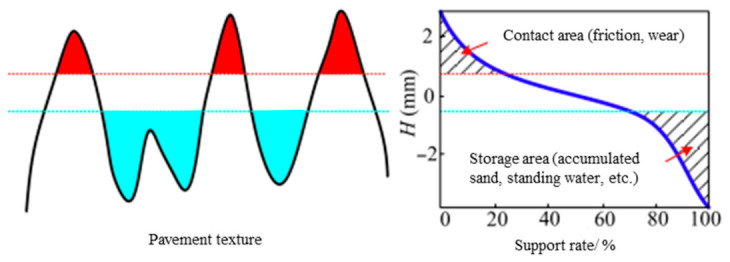
Schematic diagram of road surface texture function zoning. The red and cyan areas represent the contact area and storage area, respectively, while the corresponding dotted lines indicate their threshold levels.

**Percentage Carrying Area Height (Smr)**. As illustrated in Figure 7, the left diagram provides a schematic of the road surface texture, while the right diagram displays the bearing ratio curve, which can also be interpreted as the cumulative height probability distribution. Previous experimental studies, such as those by Yun et al. [23], have shown that the texture in the peak region of the surface usually plays a supporting role and acts as the “effective” carrier for contact (referred to as “effective contact” in this paper). Consequently, textures in this state of effective contact dominate pavement functions such as friction and wear. During the contact process, intermediate media may also act as lubricants or substances that accelerate polishing within the contact system [24].

**Texture Depth (TD)**. The average texture depth (TD) of the sample surface was determined using the manual sand-spreading method as outlined in the “Standard Test Methods of Bitumen and Bituminous Mixtures for Highway Engineering” (JTG E20-2011). The main testing procedures are as follows:

(1) Clean the rutting plate surface.
(2) Fill a 25 ± 0.15 mL sand measuring cylinder with standard sand (particle size 0.15–0.30 mm), pour the sand at the center of the rutting plate surface, and level it into a circular shape using a leveling plate.
(3) Measure the diameter of the spread circle orthogonally with a steel ruler and take the average value as the result.

(12) TD=1000Vπd24=31381d2

where TD represents the texture depth of the rutting plate surface (mm), V denotes the volume of sand (25 cm^3^), and d indicates the average diameter of the leveling plate (mm).

Self-affine fractal dimension (D). Based on the similarity between local and global characteristics of the system, the self-affine similarity reveals the fractal nature of objects. Fractal analysis quantifies the irregularity of a system or structure and assesses the similarity between its local and global characteristics. Common methods for calculating fractal dimensions include maximum likelihood estimation, periodogram methods, grid methods, and box-counting methods. This study calculated the fractal dimensions of the sample surface morphology using the box-counting method. This method involves covering the physical quantity with boxes of the same size ε when calculating the fractal dimension of the research object. The relationship between the number of boxes N(ε) and the scale ε can be evaluated by varying the box size [25]. This relationship is expressed as:

(13) $$N(\varepsilon )\propto \varepsilon^{-D}$$

For a given scale ε, the formula for calculating the spatial properties of the fractal object is:

Where A(ε) varies in meanings for different fractal objects and can be interpreted as length in one dimension, area in two dimensions, or volume in three dimensions. For consistency, it is referred to as the “mass” of the box. E corresponds to the dimension of Euclidean geometric space.

Substituting Equation (13) into Equation (14) and taking the logarithm of both sides yields:

(14) lnA(ε)=(E-D)lnε+lnC

where C is a constant. The fractal dimension D relates to the slope k in the double logarithmic coordinate system of A(ε)∼ε. Here, k can also be interpreted as the Hurst exponent. Equation (15) allows for a straightforward calculation of A(ε) for a specified value of ε. Therefore, the fractal dimension D calculated using the box-counting method is:

(15) D=E-k

## 3. Results and Discussion

### 3.1. Effective Contact Texture Analysis

**Figure 8:**
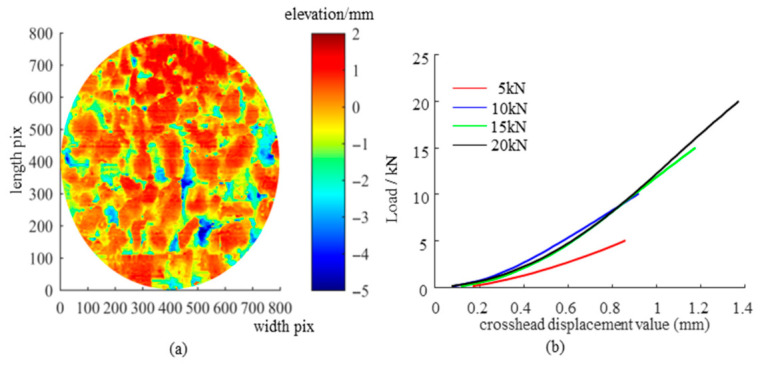
3D texture and imprint texture on the sample surface: (**a**) 3D elevation map of the sample surface; (**b**) load-displacement curves under different loads.

**Figure 9:**
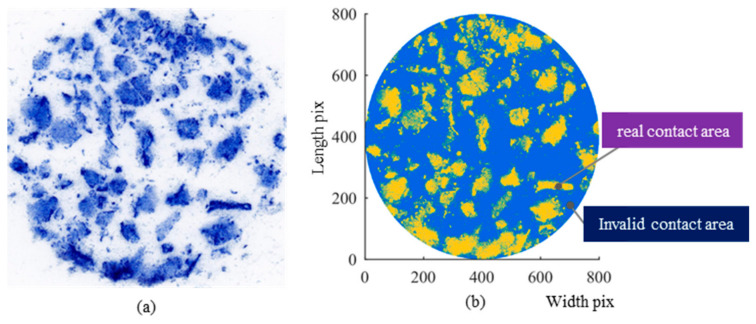
Digital images of carbon-paper imprint textures for SMA10: (**a**) Original contact imprint image; (**b**) Binary classification result of contact areas.

The 3D laser scanning system described in Section 2.2.1 was employed to obtain the surface textures of the nine samples. The surface texture of the imprint area was extracted through segmentation using the SMA10 as an example, and the resulting surface morphology from 3D reconstruction is presented in Figure 8a. The samples were subjected to loads ranging from 5 to 20 kN using the MTS system, as shown in Figure 8b. It should be noted that although a 25 kN loading condition was attempted, the paper interlayer used for imprint extraction ruptured under this condition, and a reliable contact imprint could not be obtained. Therefore, only the results from 5 to 20 kN were considered in the subsequent analysis. The texture of the contact area from the photocopied imprint was captured using a printer scanner, as illustrated in Figure 9a. Figure 9b visually indicated that the circular area represents the nominal contact area, the gold area (which corresponds to black in the binary image) signifies the real contact area, and the blue area (corresponding to white in the binary image) is considered the ineffective contact area. It is apparent that the real contact area expanded with increasing load.

**Figure 10:**
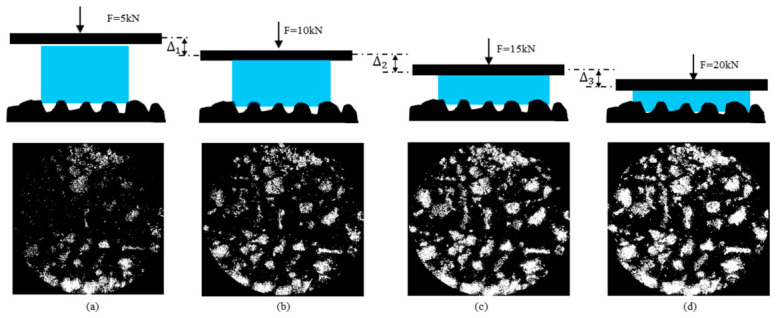
Imprint textures of rubber and asphalt mixture rutting plates under different loads (SMA10): (**a**) 5 kN; (**b**) 10 kN; (**c**) 15 kN; (**d**) 20 kN. In the schematic diagrams, the blue areas represent the rubber pads, and the black areas indicate the loading plates and asphalt mixture. In the imprint images, the white areas represent the effective contact areas, while the black areas denote the non-contact areas.

**Figure 11:**
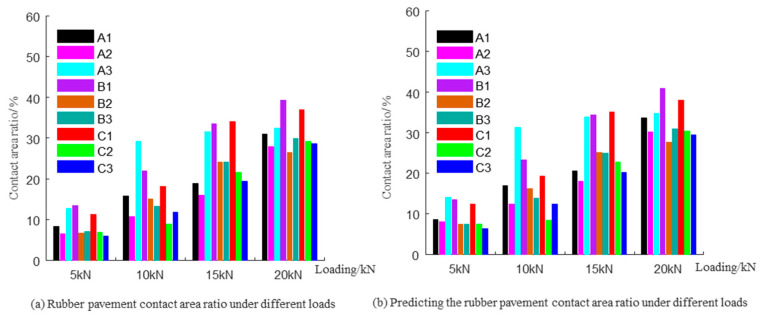
Statistics of real contact area ratios under different loads: (**a**) rubber pavement contact area ratio under different loads; (**b**) predicting the rubber pavement contact area ratio under different loads.

The scanned digital images were binarized using the maximum variance thresholding method to obtain the imprint textures under loads of 5 to 20 kN, as depicted in Figure 10a–d. Figure 11a illustrates the statistics on the real contact area ratio. As the load increased, the real contact area ratio also rose. When the load reached 20 kN, the real contact area ratios for all samples remained below 40%. This phenomenon is attributed to differences in surface voids. As the load increased, the protruding aggregate portions continued to penetrate the rubber, enhancing the interlocking contact. Due to the elastic properties of rubber, the penetration texture depth continually increased, which led to an expansion of the real contact area.

These results indicate that the rubber–pavement contact state remained highly incomplete even under relatively high loading conditions. For all tested surfaces, the real contact area ratio was still below 40% at 20 kN, demonstrating that the nominal contact area may substantially overestimate the actual interfacial area involved in friction generation. This finding is consistent with the contact-mechanics concept that real contact occurs only at limited asperity regions on rough surfaces [7].

The surface texture information of the samples was obtained using a 3D laser profilometer. The 3D texture elevation data of the contact area was extracted and imported into MATLAB (version R2024a) to compute the surface texture information for the nine samples. The support curve within the texture was calculated using the imprint area ratio, and the resulting support height Smr is detailed in Table 5. The support height data allows for the determination of the top data of the 3D texture. The area ratio of the 3D texture was obtained through “slicing segmentation” of the top data, as illustrated in Figure 11b. The results indicated that the contact area ratios calculated for the nine samples subjected to different loads were very close to the real contact area ratios, with an absolute error not exceeding 2.59%. The close agreement between the support-curve-based reconstruction and the imprint-derived contact area further confirms the feasibility of the proposed measurement framework. Rather than serving only as an image-processing procedure, this method links two-dimensional imprint information with three-dimensional pavement texture data, thereby providing a physically interpretable route for extracting effective contact parameters from the rubber–pavement interface [11].

### 3.2. Analysis of Texture Evaluation Parameters

#### 3.2.1. Comparative Analysis of TD

**Figure 12:**
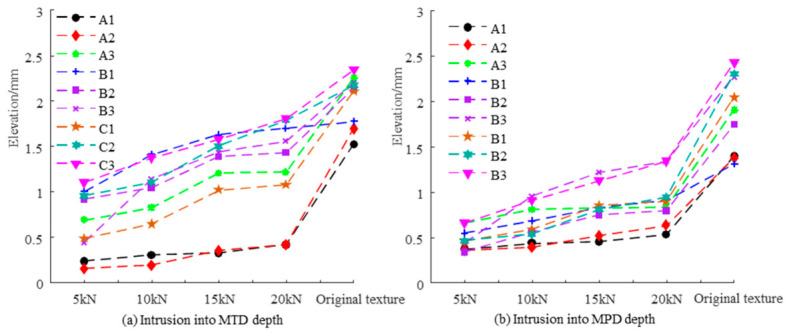
Penetration depth of rubber into the texture under different loads.

**Figure 13:**
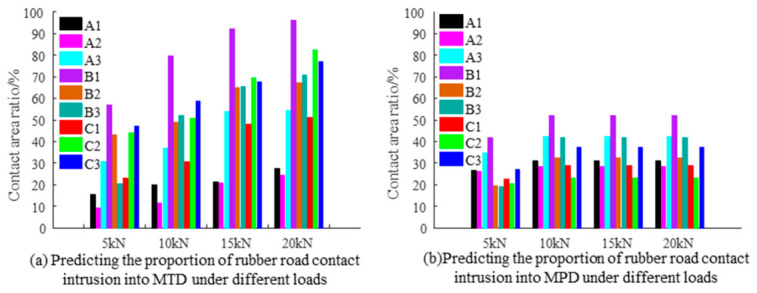
Variation of penetration depth ratios under different loads.

The calculations of TD were conducted by converting the support height from the imprint area ratio into the top data of the slices under various loads. The results yielded the statistics of MTD and MPD, as presented in Table A1 in Appendix A, as well as Figure 12a,b. It was observed that with increasing load, the penetration depth of the rubber pad into the original pavement 3D texture consistently increased. The ratio of penetration texture depth to original texture depth (where MTD and MPD values were calculated from 3D laser scanning) is displayed in Figure 13a,b. The penetration depth ratio positively correlates with the load. The SMA10 demonstrated the highest penetration depth ratio, measuring 96% for MTD and 43.6% for MPD at 20 kN. Conversely, the AC13 showed the lowest penetration depth ratio, with 24.6% for MTD and 45.7% for MPD at 20 kN.

#### 3.2.2. Comparative Analysis of Fractal Dimension

The fractal dimensions for the top data of the penetrated 3D texture and the 2D images of the imprint texture under varying loads were computed using the box-counting method, as shown in Table A2 in Appendix A. It was revealed that within the load range of 0 to 20 kN, the fractal dimension D of nearly all pavement penetration textures showed an increasing trend with load. The fractal dimension D of the top data from the penetration texture slices of AC pavement and rubber pad varied between 1.5246 and 1.8737, whereas the fractal dimension D of the contact imprints ranged from 1.4696 to 1.7319. The fractal dimension D of the top data from the penetration texture slices of SMA pavement and rubber pad ranges from 1.4678 to 1.7641, whereas the fractal dimension D of the contact imprints varies from 1.4493 to 1.7155. The fractal dimension D of the top data from the penetration texture slices of OGFC pavement and rubber pad was between 1.4947 and 1.8422, while the fractal dimension D of the contact imprints was between 1.4457 and 1.7460. The difference in fractal dimensions between penetration and imprint textures reveals an absolute error of 0.1748. This further suggests that the calculated fractal dimension D of the 2D imprint texture can effectively replace the fractal dimension D of the top slice from the 3D pavement surface texture.

### 3.3. Correlation Between Pavement TD and Skid Resistance

**Figure 14:**
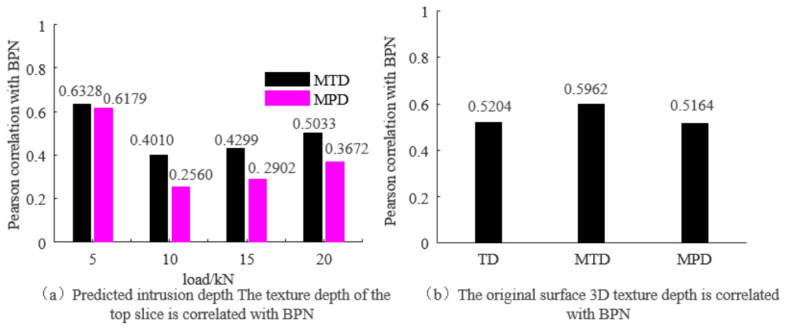
Correlation between texture index and BPN.

The initial British Pendulum Number (BPN) and texture depth (TD) of the nine asphalt mixture rutting plates were measured and are summarized in Table 6 and Table 7, respectively. Based on these basic properties, a Pearson correlation analysis was conducted between the MTD, MPD, and the BPN, as presented in Figure 14a. Both MTD- and MPD-based penetration texture parameters showed moderate correlations with BPN. At the 5 kN loading level, the correlation between MPD and BPN was slightly higher than that between MTD and BPN, with R^2^ values of 0.6328 and 0.6179, respectively. However, MTD-based penetration parameters showed relatively stable correlations across different loading levels, indicating that penetration-related texture descriptors can provide useful information for skid resistance evaluation. It is noteworthy that a load of 5 kN is equivalent to 0.64 MPa, which is close to the wheel pressure of 0.7 MPa under the standard axle load of BZZ_100. Overall, the correlation between MTD and BPN remained around 0.5 across different loads. Figure 14b illustrates the correlations between the average TD derived from the sand-spreading method, the MTD and MPD obtained through 3D laser scanning of the rutting plate, and the BPN. The correlations were all above 0.5, and the highest correlation occurred between MPD and BPN, with R^2^ = 0.5962. This further suggests that the imprint texture metrics obtained through this study are comparable to those from 3D laser scanning and can be utilized to indirectly predict pavement skid resistance. Existing studies [26] suggest that these two methods may be interchangeable in predicting pavement skid resistance. However, in this study, the indirect representation of skid resistance using MTD is more effective than that using MPD.

**Figure 15:**
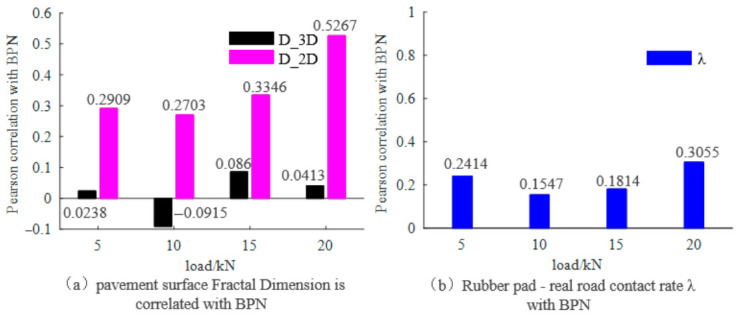
Correlations between the self-affine fractal dimension D of pavement surface, the rubber–pavement real contact rate, and the BPN.

The self-affine fractal dimensions D_3_D of the penetrated 3D textures (top slices) and D_2_D of the imprint textures were analyzed for their Pearson correlation with BPN under different loads. As indicated in Figure 15a, there was no notable correlation between D_3_D, D_2_D, and BPN. A correlation analysis was performed between the contact area ratios under different loads and the BPN values obtained from the pendulum friction tester, with results shown in Figure 15b. It was observed that the real contact area ratio did not demonstrate a significant correlation with the skid resistance. This suggests that the fractal dimension D of the texture and the real contact area ratio λ do not exhibit a straightforward linear relationship with the pavement friction factor μ.

Overall, the correlation results suggest that penetration texture-related parameters are more closely associated with skid resistance than the self-affine fractal dimension or the real contact area ratio alone. This indicates that the effective penetration and interlocking of rubber with the upper portion of pavement texture may be more functionally meaningful for skid resistance evaluation than global surface irregularity descriptors [21]. Therefore, the proposed method may serve as a useful laboratory screening tool for comparing asphalt mixtures and identifying texture features that are more directly related to pavement friction performance [22].

## 4. Limitations

This study has several limitations that should be explicitly acknowledged. First, natural rubber was used as a simplified surrogate for tire tread, and therefore the present experiments do not fully reproduce the compound formulation, tread pattern, internal inflation, or dynamic viscoelastic response of real tires. Second, the tests were conducted under static laboratory loading at room temperature, without considering speed effects, temperature variation, frictional heating, or water film conditions. Third, the method was validated on nine asphalt pavement samples only, and its applicability to other pavement types and broader service conditions still requires further verification. Fourth, a full repeatability assessment based on independent parallel tests for each pavement-load condition was not completed in the present study. Therefore, although the consistency between imprint-based and reconstruction-based results supports the feasibility of the proposed approach, the statistical repeatability of the method still requires further verification in future work. Fifth, it should be noted that a circular loading area was adopted in this study to facilitate uniform load application, reduce eccentric loading, and provide a stable configuration for methodological development. The objective of the present work was to establish a rapid measurement framework for extracting true contact parameters at the rubber–pavement interface, rather than to reproduce the full footprint geometry of an inflated tire. Therefore, the circular contact used here should be regarded as a controlled laboratory simplification. The influence of actual tire footprint shape, tread pattern, and inflation-induced pressure distribution on the measured contact response remains an important topic for future investigation. Accordingly, the present study should be regarded as a methodological and mechanistic step toward contact-based characterization rather than a complete replacement for full-scale tire–pavement friction evaluation under field conditions.

## 5. Conclusions

This study developed a rapid and non-destructive method for characterizing true rubber–asphalt pavement contact texture by combining imprint extraction, digital image processing, and three-dimensional pavement texture reconstruction. Based on the experimental results obtained from nine asphalt pavement samples under loads from 5 to 20 kN, the following conclusions can be drawn:

(1) The real contact area increased with load for all tested pavements, but the real contact area ratio remained below 40% even at 20 kN, indicating that nominal contact assumptions may overestimate the actual interface involved in friction generation.
(2) The support-curve-based method was able to reconstruct penetration-related contact texture from 3D pavement data, and the calculated contact area showed good agreement with the imprint-based results, with an absolute error not exceeding 2.59%.
(3) Penetration texture-related parameters exhibited clearer relevance to skid resistance than self-affine fractal dimension, suggesting that effective rubber penetration into the upper pavement texture is a more functionally meaningful indicator than global irregularity alone.
(4) The proposed method provides a practical laboratory framework for extracting true contact parameters and may serve as a useful supplementary tool for texture-based skid resistance evaluation and mixture screening, although further validation under more realistic tire and environmental conditions is still needed.

## Figures and Tables

**Table 1:** Main technical indicators of coarse and fine aggregates.

Test Parameters	Type1 g/m^3^	Type2 (%)	Type3 (%)	Type4 (%)	Toughness (%)
Technical standards	≥2.60	≤2.0	≤26	≤15	≤12
Test results	2.725	1.2	22.5	12	7.2

Note: Type1 represents the apparent density of coarse aggregate, Type2 represents the water absorption, Type3 represents the rate of crushing value, and Type4 represents the content of needle-like and flaky particles.

**Table 2:** Performance parameters for the rubber pads.

Test Parameters	Test Results	Standards	Test Method
Breakdown voltage (KV)	>70	≥35	GB/T 1695-2005
Tensile strength (Mpa)	20.8	≥5	GB/T 528-2009
Elongation at break (%)	528	≥350	GB/T 528-2009
Hot air aging (60 °C, 3h)			
Shore hardness (after aging) (shoreA)	52	65 ± 5	GB/T 3512-2014

Note: Equipment utilized includes a breakdown voltage tester (Beijing Aerospace, Beijing, China), a thermal aging chamber (RIUKAI, Dongguan, China), a servo-controlled tensile testing machine (Domestic Manufacturers, Jinan, China), and a hardness tester (Wance Instrument, Jinan, China).

**Table 3:** Overview of testing equipment.

Equipment and Materials	Functions and Testing Details	Specifications
MTS (MTS Systems Corporation, Eden Prairie, MN, USA)	Provide a load and record beam displacement	100 kN capacity, displacement relative error within ±0.5%
paper A4 (Double A Paper Co., Ltd., Bangkok, Thailand)	Contact the texture imprint carrier	210 × 297 × 0.28 mm (thickness)
Rubber pad (Shanghai Jingjie Rubber Pad Co., Ltd. (MAGIC PAD), Shanghai, China)	Simulate the rubber tire	See Table 2
Carbon paper (Cangnan Longgang Bee Tiger Stationery Co., Ltd., Wenzhou, China)	Provide the source for imprints	148 × 210 mm
Printer—Toshiba e-Studio2506Series (Toshiba Tec Corporation, Tokyo, Japan)	Extract 2D texture imprint digital images	Digital image resolution of 300 dpi
Rutting plate (Jinan Luda Testing Equipment Co., Ltd., Jinan, China)	Simulate the pavement surface	300 × 300 × 50 mm

Note: (1) The MTS, as a load application device, effectively ensures load accuracy and prevents eccentric loading. (2) Carbon paper is more cost-effective and accessible than pressure-sensitive films. Its thickness of only 0.28 mm minimizes disturbances in the contact between the rubber pad and the pavement surface.

**Table 4:** Converted load values for overpressure test.

Applied Force (kN)	Converted Load (MPa)
5	0.64
10	1.27
15	1.91
20	2.55
25	3.18

Note: although the 25 kN loading level was attempted during testing, its result was not included in the final analysis because the paper interlayer ruptured and a reliable imprint could not be obtained.

**Table 5:** Support height of 3D texture under different loads.

Sample ID	Support Height Under Different Loads (mm)				
	5 kN	10 kN	15 kN	20 kN	Original Texture
A1	0.2365	0.3044	0.364	0.4192	1.5219
A2	0.1545	0.1915	0.3518	0.4151	1.6895
A3	0.6872	0.8263	1.2074	1.2200	2.2538
B1	1.0035	1.4075	1.6306	1.6982	1.7698
B2	0.3417	0.5593	0.7486	0.9041	1.3054
B3	0.4443	1.1393	1.4365	1.5527	2.1989
C1	0.4855	0.6475	1.0154	1.0787	2.1077
C2	0.9550	1.1009	1.5057	1.7838	2.1700
C3	1.1018	1.3714	1.5788	1.8050	2.3420

Note: A1–A3: AC_10_13_16; B1–B3: SMA_10_13_16; C1–C3: OGFC_10_13_16.

**Table 6:** BPN test for rutting plates.

Model of Rutting Board	AC10	AC13	AC16	SMA10	SMA13	SMA16	OGFC10	OCFC13	OGFC16
BPN value	69.46	60.40	57.26	63.58	61.88	67.40	64.60	54.64	50.28

**Table 7:** TD test for rutting plates.

Model of Rutting Board	AC10	AC13	AC16	SMA10	SMA13	SMA16	OGFC10	OCFC13	OGFC16
TD (mm)	0.96	1.51	1.32	1.74	2.64	3.30	3.16	3.24	4.79

## Data Availability

The original contributions presented in this study are included in the article. Further inquiries can be directed to the corresponding author.

## Appendix A

**Table A1:** Predicted 3D penetration texture depths under different loads.

Sample ID	Type	TD (mm)				
		5 kN	10 kN	15 kN	20 kN	Original Texture
A1	3D textureMTD__2D_	0.2365	0.3044	0.3640	0.4192	1.5219
	3D textureMPD__2D_	0.3697	0.4388	0.4557	0.5332	1.3949
A2	3D textureMTD__2D_	0.1545	0.1915	0.3518	0.4151	1.6895
	3D textureMPD__2D_	0.3595	0.3915	0.5211	0.6287	1.3761
A3	3D textureMTD__2D_	0.6872	0.8263	1.2074	1.2200	2.2538
	3D textureMPD__2D_	0.6548	0.8051	0.8226	0.8292	1.9013
B1	3D textureMTD__2D_	1.0035	1.4075	1.6306	1.6982	1.7698
	3D textureMPD__2D_	0.5443	0.6766	0.8260	0.9041	1.3054
B2	3D textureMTD__2D_	0.9176	1.0422	1.3866	1.4289	2.1325
	3D textureMPD__2D_	0.3417	0.5593	0.7486	0.7952	1.7385
B3	3D textureMTD__2D_	0.4443	1.1393	1.4365	1.5527	2.1989
	3D textureMPD__2D_	0.4347	0.9503	1.2100	1.3309	2.2695
C1	3D texture MTD__2D_	0.4855	0.6475	1.0154	1.0787	2.1077
	3D textureMPD__2D_	0.4576	0.5874	0.8494	0.8948	2.0308
C2	3D textureMTD__2D_	0.9550	1.1009	1.5057	1.7838	2.1700
	3D texture MPD__2D_	0.4654	0.5336	0.8030	0.9392	2.2987
C3	3D texture MTD__2D_	1.1018	1.3714	1.5788	1.8050	2.3420
	3D texture MPD__2D_	0.6556	0.9036	1.1198	1.3366	2.4239

Note: A1–A3: AC_10_13_16; B1–B3: SMA_10_13_16; C1–C3: OGFC_10_13_16.

**Table A2:** Fractal dimensions of 3D textures and contact imprints under different loads.

Sample ID	Type	TD (mm)			
		5 kN	10 kN	15 kN	20 KN
A1	3D texture	1.5246	1.5279	1.6538	1.7379
	Contact imprint	1.5014	1.6217	1.6636	1.7319
	Absolute error	0.0232	0.0938	0.0098	0.0060
A2	3D texture	1.5481	1.6093	1.6618	1.7338
	Contact imprint	1.4696	1.5519	1.6060	1.6815
	Absolute error	0.0785	0.0574	0.0558	0.0523
A3	3D texture	1.6695	1.7453	1.7558	1.8737
	Contact imprint	1.5581	1.7085	1.7039	1.6989
	Absolute error	0.0225	0.0368	0.0519	0.1748
B1	3D texture	1.6035	1.6876	1.7444	1.7641
	Contact imprint	1.5821	1.6428	1.7009	1.7155
	Absolute error	0.0214	0.0448	0.0435	0.0486
B2	3D texture	1.4678	1.5899	1.6672	1.6836
	Contact imprint	1.4493	1.5777	1.6591	1.6465
	Absolute error	0.0185	0.0122	0.0081	0.0371
B3	3D texture	1.5293	1.6192	1.7015	1.7291
	Contact imprint	1.4735	1.5578	1.6499	1.6719
	Absolute error	0.0558	0.0614	0.0516	0.0572
C1	3D texture	1.6149	1.6743	1.7524	1.8422
	Contact imprint	1.5125	1.5948	1.6792	1.7460
	Absolute error	0.1024	0.0795	0.0732	0.0962
C2	3D texture	1.4947	1.5292	1.6589	1.7007
	Contact imprint	1.4457	1.5040	1.6536	1.6715
	Absolute error	0.0490	0.0252	0.0053	0.0282
C3	3D texture	1.5296	1.6172	1.6804	1.7317
	Contact imprint	1.4535	1.5451	1.6122	1.6603
	Absolute error	0.0761	0.0721	0.0682	0.0714

Note: A1–A3: AC_10_13_16; B1–B3: SMA_10_13_16; C1–C3: OGFC_10_13_16.
